# Comparing paper Letters in addition to Emailed Audit and feedback in Refining Asthma treatment to Improve clinical and environmental Results in primary care through a cluster randomised controlled trial: the CLEAR AIR study

**DOI:** 10.1136/bmjresp-2025-003601

**Published:** 2026-04-17

**Authors:** Owen Thomas, Bethan Copsey, Paul Carder, Imran Mohammed, Stella Johnson, Katherine Hickman, Toby Capstick, Robbie Foy, Sarah Alderson

**Affiliations:** 1 Leeds Institute of Health Science, University of Leeds, Leeds, UK; 2 School of Medicine, University of Leeds, Leeds, UK; 3 West Yorkshire Research and Development Team, West Yorkshire Integrated Care Board, Wakefield, UK; 4 Respiratory Lead, West Yorkshire Integrated Care Board, Wakefield, UK; 5 Medicines Management and Pharmacy Services, Leeds Teaching Hospitals NHS Trust, Leeds, UK

**Keywords:** Asthma, Asthma Guidelines, Asthma in primary care, Asthma Pharmacology, Clinical Epidemiology, Inhaler devices

## Abstract

**Background:**

Suboptimal use of preventer inhalers and salbutamol reliever overprescribing are associated with preventable asthma deaths and are a major source of primary care carbon emissions. Audit and feedback produces modest behaviour change by assessing clinical performance and delivering feedback to encourage improvement. Although feedback is increasingly delivered digitally, clinicians may respond more to additional printed feedback reports. We evaluated whether combined digital and paper feedback was more effective than digital-only feedback in promoting safer and greener asthma prescribing at the practice level.

**Methods:**

In this parallel, cluster randomised controlled trial, all 273 primary care practices in West Yorkshire were assigned within their primary care network clusters by stratified, permuted block randomisation to receive seven bimonthly reports on asthma prescribing either in ‘digital and paper’ (intervention) or ‘digital-only’ (control) formats. The primary outcome was the proportion of preventer inhalers prescribed in pressurised metred-dose devices due to their high carbon footprint. Intervention group allocation was concealed. The intention-to-treat population was analysed and adjusted for both potential confounders and preintervention achievement.

**Results:**

Final analysis assessed 270 practices in 26 clusters per arm due to practice mergers within the control group. There was no significant difference between the intervention groups based on change in the primary outcome (intervention—0.15%; control—0.19%; risk ratio—1.00; 95% CI 0.98 to 1.03) nor any secondary outcome. Analysis of both interventions combined showed a background trend of mixed improvement following feedback.

**Conclusions:**

There was no evidence that combined paper and digital feedback was more effective than digital-only feedback, despite the background of mixed improvements following both interventions. Challenges remain to understanding the barriers to influencing the prescribing of preventer inhalers and transitioning inhaler devices towards low-carbon ‘green’ alternatives; however, this study demonstrated the value of an efficient ‘real-world’ trial embedded within an existing quality improvement initiative.

**Trial registration number:**

NCT05761873.

**WHAT IS ALREADY KNOWN ON THIS TOPIC**

- Suboptimal use of preventer inhalers and salbutamol reliever overprescribing leads to poorer outcomes in patients with asthma. Audit and feedback (A&F) generally has modest effects on clinician prescribing behaviour, but the impact of paper feedback reports in addition to digital reports was uncertain.

**WHAT THIS STUDY ADDS**

- Against a background of variable improvements in prescribing after an A&F intervention, the addition of paper to digital feedback reports did not produce further change in prescribing patterns within primary care.

**HOW THIS STUDY MIGHT AFFECT RESEARCH, PRACTICE OR POLICY**

- Adding paper to digital feedback reports appears to offer no gains in effectiveness and adds to the burden of emissions already produced by high-carbon devices. This study illustrated the value of efficient, pragmatic randomised trials in answering specific questions about how to promote the implementation of evidence-based practice. Further research is needed to understand the barriers influencing the prescribing of preventer inhalers and persistent use of high-carbon inhaler devices.

## Introduction

 Accumulating evidence indicates that climate change is detrimental to respiratory health. This harm is driven by changes in environmental allergen levels, new and higher levels of aerosol chemical irritants and particulate matter, the carcinogenic nature of airborne chemicals and the direct aggravation of chronic respiratory disease by pollutants.^1^ In a recent landmark case, a UK coroner concluded that the death of a young child with asthma was directly linked to vehicle air pollution.^2^

Asthma is a common long-term condition that is in the top 20 causes of mortality in those under 9 years of age and causes 457 000 deaths per year worldwide.^3^ Poor asthma care has been found to play a significant role in two-thirds of UK asthma-related deaths, with suboptimal use of preventer inhalers and short-acting beta agonist (SABA) reliever overprescribing being particularly risky.^4^ Lack of preventer inhaler use is strongly associated with asthma morbidity and mortality. In the UK National Review of Asthma Deaths, 38% of deaths occurred in patients who had received three or fewer preventer prescriptions in the preceding year, while a large US cohort found a 21% reduction in asthma mortality with each additional preventer inhaler dispensed.^5 6^ Optimal drug delivery with pressurised metred-dose inhalers (pMDIs) requires coordinated actuation and inhalation, a skill that is frequently impaired in children, older adults and those using multiple devices; spacers can mitigate this but are bulky and require specific monthly maintenance.^7 8^ By contrast, some dry powder inhalers (DPIs) achieve up to two times the lung deposition of comparable pMDIs, and correct DPI technique has been observed in nearly two times as many patients, with better retention over time.^9^,^11^ Real-world evidence from the Salford lung study—a large, open-labelled randomised controlled trial conducted in routine clinical practice—suggests that DPIs may also improve outcomes compared with pMDIs and, in the UK, offer broader anti-inflammatory reliever and maintenance and reliever therapy (MART) licensing; no pMDI is licensed for MART in patients under 18.^12 13^ While pMDIs have a role in asthma management and the correct inhaler is the one that suits the patient, the continued disproportionate over-reliance on pMDIs in England goes beyond this principle, often excluding devices that may be clinically preferable.^14 15^ Regular SABA inhaler overuse can increase eosinophilic inflammation, increase bronchial hyper-responsiveness, downregulate respiratory beta-2-receptors and promote tolerance to its beneficial clinical effects.^16 17^ The use of three or more SABAs per year is associated with an increased risk of exacerbations, while using 12 or more is associated with an increased risk of death.^18 19^

In the UK, 13% of the primary care carbon footprint comes directly from pMDIs containing hydrofluorocarbon (HFA)-134a and HFA-227 high-carbon propellants.^20^ This creates an unsettling paradox linking some asthma treatments directly to the pollutants that exacerbate asthma. Recent national and international guidelines have recommended that asthma treatment avoids SABA-only management and high-carbon emitting inhaler devices in favour of regimens that focus on combination ‘green’ low-carbon inhaler devices, such as DPIs that contain inhaled corticosteroids (ICS) and long-acting beta agonists.^13 21^

Audit and feedback (A&F) is an established quality improvement method that involves providing summaries of performance to a target individual or group to motivate changes in practice.^22^ A recent Cochrane review has demonstrated that A&F changes clinical behaviour and is particularly effective when targeting prescribing practices with low baseline compliance.^23^ Despite this, there is considerable scope for optimising A&F effectiveness by testing different methods of feedback delivery.^24^

Earlier research in general practice across West Yorkshire (UK) has demonstrated the benefits of A&F in reducing potentially harmful prescribing.^25^,^27^ Qualitative work from the same setting suggested that clinicians may engage more effectively with posted paper copies of feedback reports than with emailed digital versions, as physical documents enhance visibility, stimulate discussion and prompt action during in-person meetings.^28^ Although few healthcare studies have compared the behavioural impact of digital versus paper feedback on professionals, this question has been widely examined in psychology and education. A recent meta-analysis reported that students reading printed text consistently achieved higher comprehension scores than those reading the same material on screens.^29^ Proposed mechanisms include the ‘shallowing effect’, whereby digital media—such as emails—encourages rapid, surface-level information processing and a browsing mindset, reducing reflection and comprehension relative to paper-based resources.^30^

However, printing and posting paper reports at scale carries substantial environmental and financial costs.^31^ The West Yorkshire National Health Service (NHS) Integrated Care Board (WY ICB) had identified digital transformation as a means to improve patient outcomes while supporting environmental sustainability.^30^ Against this backdrop, the present study examined whether supplementing digital A&F with paper reports improved the quality and sustainability of asthma inhaler prescribing in primary care, which could offset the economic and carbon impact of producing and distributing paper reports.

## Methods

### Design

This study used a two-arm, parallel, cluster randomised controlled trial (cRCT) design with primary care practices as the unit of analysis.

### Interventions

Primary care practices were randomly assigned to receive seven evidence-based bimonthly asthma prescribing reports either via email and post (intervention), or email alone (control) over a 12-month period. Reports contained audited information on individual practice prescribing of the seven study outcomes, compared with both a fixed standard and anonymised comparisons of other practices. These reports included evidence- and theory-informed content based on previous research (see online supplemental appendices I and II for a sample report and a template for intervention description and replication (TIDieR) summary of the intervention).^27 32 33^ Each report contained accompanying educational material written jointly by the research team and local medicines optimisation leads. The reports also included suggested action plans for practices.

### Outcomes

The primary outcome was the number of ‘high-global warming potential’ preventer pMDI inhalers prescribed as a proportion of the total prescribed preventer inhalers for patients with asthma from each primary care practice—limited to those aged over 5. While there is a variation in pMDI inhalers in relation to their carbon footprint, for this analysis, all pMDI inhalers were considered as being of ‘*high-global warming potential’*. Secondary outcomes included the proportion of patients with asthma that were: using 6 or more SABAs per year; using 12 or more SABAs per year; using 3 or fewer ICS inhalers per year; requiring 2 or more oral courses of prednisolone per year; aged 0–19 without smoking exposure status recorded and using a mix of inhaler device types (patients using similar inhaler devices have better asthma control and less exacerbations than those using mixed DPI/pMDI devices^34^).

### Setting

This study took place in primary care practices within West Yorkshire, UK. The region has a population of over 2.3 million, with over 20% living within the lowest decile of deprivation in England.^35 36^ The prevalences of chronic obstructive pulmonary disease (COPD) and asthma in West Yorkshire were 0.3% and 0.5% higher than the national average, respectively, in 2021 (COPD 2.2% vs 1.9% and asthma 6.9% vs 6.4%).^37^

### Recruitment, allocation and blinding

All primary care practices within West Yorkshire were informed of the trial by email and offered the opportunity to opt out. Formal signed consent was considered disproportionately burdensome, given the low risk of the intervention following the precedent of previous studies.^38^

Practices were allocated to an intervention group within clusters based on local consortiums known as primary care networks (PCNs), which were chosen to prevent contamination effects from shared access to pharmacist-led medication reviews.^39^ To maintain allocation concealment, colleagues from the WY ICB created a pseudonymised list of practices and PCNs, which were then randomly allocated to intervention or control by the research team after recruitment. A permuted block randomisation based on clusters and stratified by geographical region was completed by the primary researcher to produce a 1:1 allocation to control or intervention groups based on practice pseudonyms (this research remained blind to allocation). During the trial, it was impossible for practices to remain blinded while receiving the intervention; however, researcher blinding continued throughout.

### Power calculations

As the sample size was limited by the total number of practices within the region, power calculations were undertaken based on 273 practices forming 52 PCN clusters being allocated to two equal groups, with a mean of 5.25 practices per cluster. Baseline data gave the average number of preventer inhalers per month as 435 per practice (50% high-carbon devices). An intraclass correlation coefficient for individual practices was assumed at 0.008, based on a previous intervention study within this population.^32^ The study was estimated to have a 97% power to detect a 5% difference (50% vs 45%) at a 5% significance level after adjustment for clustering.

### Data collection

Trial data were collated, aggregated to the practice level, pseudonymised by WY ICB, then shared with the researchers at study initiation and at endpoint. The routinely recorded data were extracted from primary care electronic health record systems (System1 and EMIS) by WY ICB medicines optimisation team via structured, coded clinical system queries, as well as freely available data from OpenPrescribing.net; these data were also used to create the A&F reports.^40^ Data on practice-level demographics were collected from freely available data from the UK Government National General Practice Profiles.^41^ No data on individual prescribers or individual patients were available to the research team.

### Patient and public involvement and engagement (PPIE)

While there was no direct PPIE in the implementation of this study, its design follows on from collaborative work among WY ICB, the National Institute for Health and Care Research (NIHR) Yorkshire and Humber Applied Research Collaboration and the University of Leeds to improve the effectiveness of A&F for high-priority prescribing issues. This research group has an established specialised implementation research PPIE panel comprised of eight people from diverse ethnic, occupational and social backgrounds with considerable collective lay experience in healthcare governance, national audits, patient advocacy and community development. Discussions from this group included wider conversations about the impact of implementation research on patients and the public, as well as challenging the research team to find efficient ways to improve the effectiveness of A&F for topics of high priority and where the potential for patient benefit is greatest. This panel has now been running for over a decade and has fed into the work that helped design this study.

### Analysis

Practices that had merged during the study period had their baseline measurements merged for analysis. Where multiple practices merged, all practices had been allocated to the same arm; therefore, the merged practice was given the same allocation. This will be shown through an adapted Consolidated Standards of Reporting Trials (CONSORT) diagram.^42^ Analysis of the primary outcome was undertaken using an intention-to-treat modality and modelled on Stata 18.^43^ A generalised estimating equations (GEE) model with a Kauermann–Carroll bias correction was used to calculate a risk ratio that assessed if there was a difference in the primary outcome between intervention and control groups over the study period. This was adjusted for relevant covariates, stratification and clustering effects. This method was also used for secondary outcomes presented as proportions, while a generalised linear mixed model (GLMM) using a restricted maximum likelihood (REML) estimation was used to assess continuous outcomes (online supplemental appendix III). A sensitivity analysis was planned, involving calculating GEE ORs and a GLMM REML mean difference for all outcomes presented as proportions, alongside all model calculations being rerun with the exclusion of any merged observations (online supplemental appendix IV).

To assess the overall impact of the intervention compared with baseline, both treatment arms were combined to undertake a before–after analysis using the Wilcoxon matched-pairs signed-rank test on all outcomes. A sensitivity analysis was undertaken by reanalysing the data with the exclusion of any merged observations.

### Trial registration and ethical approval

This trial was registered with clinicaltrials.gov (NCT05761873) and had ethical approval from the University of Leeds (MREC 22-063) and the NHS Health Research Authority (IRAS 321442). This study has been reported to the CONSORT checklist and the cRCT CONSORT extension (online supplemental appendix V).

## Results

**Figure 1:**
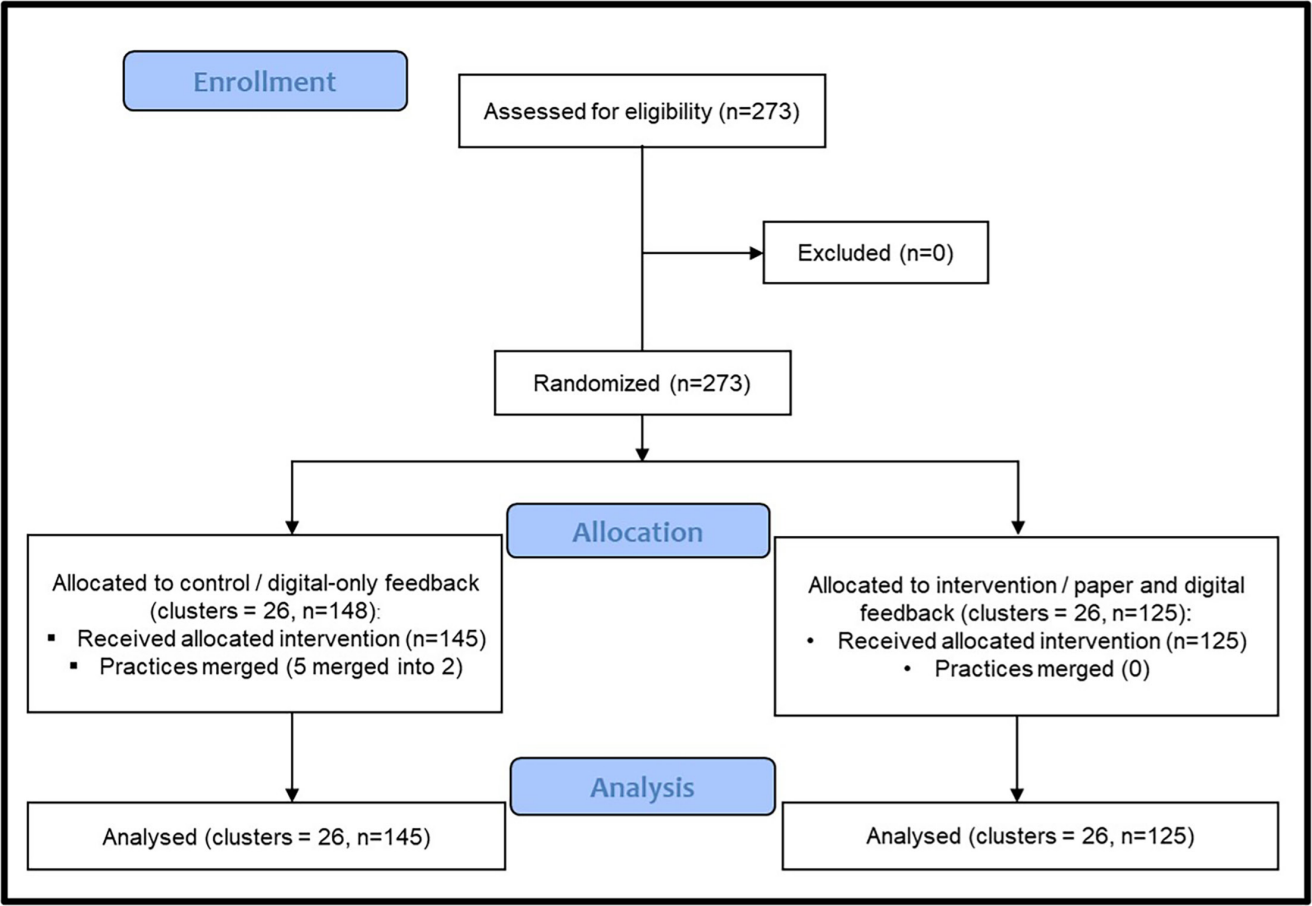
CONSORT flow diagram adapted from Hopewell *et al*.^42^ CONSORT, Consolidated Standards of Reporting Trials.

From May 2023 to May 2024, 273 practices received the asthma feedback reports. While no practices withdrew from the trial, 2 practices and 3 practices merged, respectively, to form 2 new practices, leaving 270 practices for analysis. All merged practices were from the control arm and no practices changed intervention allocation during the trial (figure 1). The median practice population index of multiple deprivation was in the third decile and the mean asthma prevalence was 6.25% (table 1). Preintervention, the mean proportion of patients using pMDI preventer devices was 50.77%, with 38.79% of patients using at least three preventer inhalers per year, 19.15% using six or more SABA per year and 9.07% using two or more courses of oral prednisolone per year (a proxy marker of frequent asthma exacerbation).

**Table 1:** Baseline practice variable

Baseline demographic indicator (per GP practice—n=270 practices)	Median (IQR)
Practice population size	8808 (5760–12 300)
IMD decile (decile 10=least deprived)	3 (1–5)
Number of patients with asthma	572 (330–802)
Number of patients with asthma aged 0–19	82 (51–124)
Number of preventer inhalers issued per month	378.5 (227–579)
Percentage of patients using a high-carbon pMDI preventer device	50.21% (44.85%–56.03%)
Percentage of patients using six or more SABA per year	17.75% (12.05%–25.30%)
Percentage of patients using 12 or more SABA per year	2.49% (1.23%–4.63%)
Percentage of patients using three or more ICS per year	39.18% (34.35%–43.15%)
Percentage of patients using two or more oral prednisolone courses per year	8.92% (7.00%–10.93%)
Percentage of patients aged 0–19 without smoking exposure status recorded	48.85% (35.42%–70.09%)
Percentage of patients using two or more mixed inhaler devices	13.44% (10.73%–17.17%)
SABA emissions per practice (ekgCO2)	6642.85 (4071.6–9885.3)

Baseline study variables by intervention group	Digital-only (clusters=26; n=145)—Median (IQR)	Paper and digital (clusters=26; n=125)—Median (IQR)
Cluster (PCN) population size	56 431 (46 780–68 329)	48 510 (42 731–60 054)
Number of practices per cluster (PCN)	7 (4–8)	5 (4–6)
Practice population size	8802 (5386–11 782)	8814 (6236–13 245)
Practice IMD decile	2 (1–5)	3 (2–5)
Number of patients with asthma per GP practice	551 (306–813)	589 (391–772)
Number of patients with asthma aged 0–19 per GP practice	81 (51–126)	85 (51–119)
Number of preventer inhalers issued per month per GP practice	380 (202–589)	377 (262–566)

ekgCO2, estimated kilograms of carbon dioxide equivalent; ICS, inhaled corticosteroids; IMD, index of multiple deprivation; PCN, primary care network; pMDI, pressurised metred-dose inhaler; SABA, short-acting beta agonist.

Randomisation produced two intervention groups containing 26 clusters each, with 145 practices and 125 practices within the control group and intervention group, respectively. On breakdown of demographics by intervention allocation, no concerning differences were identified (table 1).

### Paper and digital feedback versus digital-only feedback

**Figure 2:**
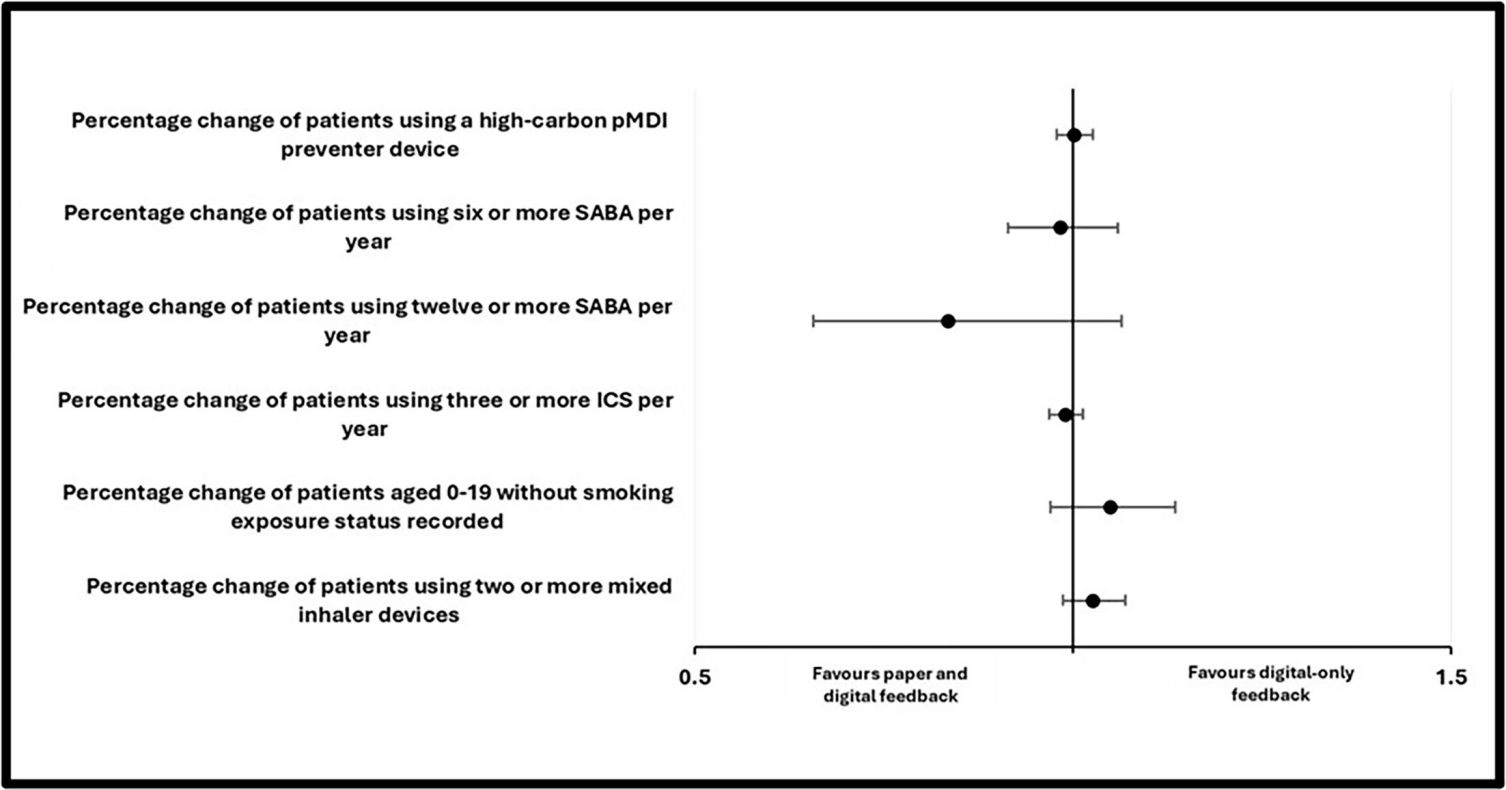
Forest plot of GEE RRs and CIs. GEE, generalised estimating equation; ICS, inhaled corticosteroids; pMDI, pressurised metred-dose inhalers; RR, risk ratio; SABA, short-acting beta agonist.

When adjusted for practice size, deprivation, baseline performance, region and clustering effects, paper and digital feedback showed no significant observed improvement compared with digital-only feedback for the primary outcome of the number of ‘high-global warming potential’ preventer pMDI inhalers prescribed as a proportion of the total prescribed preventer inhalers for patients with asthma from each primary care practice—limited to those aged over 5. There were also no statistically significant differences between the arms across any secondary outcome indicator (table 2; figure 2). Sensitivity analysis using the GEE ORs, REML GLMM calculations and full reanalysis, excluding merged practices (where the mean practice per cluster reduced to 5.19 from 5.25 with no change in PCN cluster numbers), did not meaningfully impact the statistical significance of the results or the power of the study to detect the effect sizes of significance (online supplemental appendix IV).

**Table 2:** The median absolute percentage reduction in prevalence of study outcome variables in reference to baseline performance per intervention arm

Outcome variable	Digital-only (clusters=26; n=145)—Median (IQR)	Paper and digital (clusters=26; n=125)—Median (IQR)	GEE (p-value)	GEE RR (CI)
Percentage change in patients using a high-carbon pMDI preventer device	−0.19% (−2.64%–2.02%)	−0.15% (−2.68%–2.65%)	0.868	1.00 (0.98 to 1.03)
Percentage change in patients using six or more SABA per year	−1.29% (−2.98%–0.29%)	−1.30% (−2.86% to −0.03%)	0.676	0.98 (0.92 to 1.06)
Percentage change in patients using 12 or more SABA per year	−0.34% (−1.07%–0.12%)	−0.35% (−1.13%–0.13%)	0.143	0.84 (0.66 to 1.06)
Percentage change in patients using three or more ICS per year	−0.06% (−1.74%–1.46%)	0.39% (−1.36%–1.98%)	0.401	0.99 (0.97 to 1.01)
Percentage change in patients using two or more oral prednisolone courses per year*	−0.29% (−1.34%–0.58%)	−0.58% (−1.58%–0.41%)	0.934	MD=−0.01 (−0.4 to 0.4)†
Percentage change in patients aged 0–19 without smoking exposure status recorded	−1.91% (−9.04%–12.31%)	0.03% (−11.37%–10.31%)	0.216	1.05 (0.97 to 1.13)
Percentage change in patients using two or more mixed inhaler devices	0.31% (−0.94%–1.19%)	−0.09% (−1.19%–1.17%)	0.179	1.02 (0.99 to 1.07)
Change in SABA emission per month (ekgCO2)*	−717.42 (−1576.55 to −208.90)	−750.96 (−1400 to −270.03)	0.350	IMD=−184.39 (−577.18 to 208.40)†

* Statistics for this outcome were performed by the REML GLMM.

† Mean difference in proportion (associated CIs).

ekgCO2, estimated kilograms of carbon dioxide equivalent; GEE, generalised estimating equation; GLMM, generalised linear mixed model; ICS, inhaled corticosteroids; IMD, index of multiple deprivation; pMDI, pressurised metred-dose inhaler; REML, restricted maximum likelihood; RR, risk ratio; SABA, short-acting beta agonist.

### Before–after analysis of both trial arms combined

For this secondary analysis, compared with baseline, both intervention arms combined (paper and digital and digital-only) were associated with a statistically significant reduction of 1.29% in the median percentage of patients prescribed six or more SABAs per year (table 3). There was also an estimated reduction in SABA-related median emissions of 747.84 kgCO2e per month per practice, a reduction in the median percentage of patients prescribed 12 or more SABAs per year by 0.34% and a reduction of median percentage of patients using two or more oral prednisolone courses per year of 0.42%. Despite this, there was no significant observed improvement in the primary outcome of the percentage of patients using a high-carbon pMDI preventer device, alongside three other secondary indicators (table 3).

**Table 3:** The median absolute percentage reduction for all patients in prevalence of outcome variables for both intervention groups combined, in reference to baseline performance

Percentage change in outcome variable	Median (IQR)	Wilcoxon signed-rank test (p-value)
Patients using a high-carbon pMDI preventer device	−0.16% (−2.68% to −2.29%)	0.3101
Patients using six or more SABA per year	−1.29% (−2.98%–0.19%)	0.0000
Patients using 12 or more SABA per year	−0.34% (−1.10%–0.13%)	0.0000
Patients using three or more ICS per year	0.08% (−1.67%–1.67%)	0.6956
Patients using two or more oral prednisolone courses per year	−0.42% (−1.45%–0.58%)	0.0000
Patients aged 0–19 without smoking exposure status recorded	−0.58% (−9.86%–11.63%)	0.8466
Patients using two or more mixed inhaler devices	0.07% (−1.08%–1.19%)	0.5382
SABA emission per month (ekgCO2)	−747.84 (−1532.14 to −212.03)	0.0000

ekgCO2, estimated kilograms of carbon dioxide equivalent; ICS, inhaled corticosteroids; pMDI, pressurised metred-dose inhaler; SABA, short-acting beta agonist.

## Discussion

Combined paper and digital feedback was not shown to be more effective than digital feedback alone in improving prescribing for asthma in primary care. This is an important finding as data from WY ICB and Royal Mail^44^ suggest that eliminating paper reports in similar implementation projects could directly reduce carbon emissions by approximately 1742 ekgCO₂ annually and save £16 156.43 in absolute project costs (see online supplemental appendix VI).

A secondary combined analysis of prescribing behaviour across all included practices before and after the trial revealed a heterogeneous background trend following A&F, with changes ranging from no significant shift in the proportion of high-carbon pMDI preventer prescribing to an estimated 1.29% reduction in the proportion of patients using excessive SABA reliever inhalers. However, as these results stem from a simple, before–after analysis, they cannot establish causality or exclude the influence of concurrent system-level or temporal factors. Considerable scope was found for improving asthma care, given that prior to the study approximately one in five patients with asthma used excessive SABA medication, half used high-carbon preventer devices and one in ten used frequent treatment for asthma exacerbations.

This pragmatic and efficient randomised controlled trial addressed a specific but important question on how best to deliver feedback.^28^ The study has been strengthened by using a highly repeatable set of interventions that have been described in detail and that have evidence- and theory-informed designs. The inclusion of all NHS General Practitioner (GP) practices within the West Yorkshire region, the blinding of the research team to treatment allocations throughout the study and the clustering of practices working in PCNs that might share resources, such as pharmacy teams, have significantly reduced the impact of multiple sources of bias.

Despite these strengths, this study has several limitations. Clustering can reduce the potential power of a study to show small effects. The lack of patient-level data can potentially exaggerate the results from small practices compared with larger ones, while the cross-sectional nature of the data prevents the study from adjusting for patient-level confounders, comparing absolute changes and exploring wider downstream clinical endpoints. A 6-month overlap with the ‘Leeds MART project’—aimed at switching asthma management prescribing regimes to combined MART inhalers—occurred for 20 practices within 3 Leeds PCNs between March and December 2023.^45^ Due to the pseudonymised nature of our data, it has not been possible to confirm the impact of this on the results presented here through sensitivity analysis. Alongside this, all practices recruited within the study come from primary care in the West Yorkshire region, which may limit the generalisability of the results to other locations. A 12-month follow-up may not have been sufficient to capture prescribing behaviour changes associated with annual reviews. Nevertheless, implementation trials for chronic conditions with comparable characteristics have demonstrated measurable outcome changes within this timeframe.^32 46^ More broadly, this trial took place in a high-income country that has moved significantly in recent years towards digitalising healthcare; given this context, these findings should be applied cautiously to lower income countries and healthcare systems that rely less on digital communication. The potential impacts of prevailing trends and external influences cannot be excluded or accounted for within the secondary, combined before–after analysis used to assess the impact of the combined underlying A&F intervention. However, their inclusion helps to provide context and allow hypothesis generation when comparing the different outcome variables.

A Cochrane review of printed educational materials in healthcare settings prior to 2019 found that the vast majority of the literature focused on comparing paper materials to no intervention; a meta-analysis concluded that these printed materials are likely to improve the practice of healthcare professionals, especially when dichotomous decisions for these professionals are the target of intervention.^47^ Only one randomised study was identified within this review that focused on comparisons between digital and printed materials, which identified no difference between the interventions but with a low level of certainty.^47^ In the post-COVID-19 era, many healthcare systems have rapidly transitioned from analogue and paper ways of working to fully digitalised platforms out of necessity.^48 49^ This may partly explain why the qualitative evidence suggesting preferences for paper reports did not translate into increased effectiveness in our trial, as clinicians and services—having rapidly adapted to digital workflows during the pandemic—may now be more accustomed to, efficient with and reliant on digital platforms, reducing the relative advantage that paper-based materials once held.^28^

Comprehensive work has already established convincing evidence of the reproducible effectiveness of A&F in healthcare, which is particularly reliable when it is targeted specifically at prescribing practices with low baseline compliance.^50^ Despite the limitations of before–after analysis without controls, results of the changes to prescribing behaviours overall were included to provide important context. While reductions in both SABA overprescribing and mean SABA-related carbon emissions were observed following this implementation project, it is notable that prescribing of preventer inhalers and high-carbon pMDI devices remained unchanged. Recent guideline updates identify SABA overprescribing as a marker of poor asthma management, and elements of our intervention—such as improving preventer adherence, optimising therapy and documenting risk factors—may have reduced SABA reliance indirectly without requiring specific SABA-focused prescribing changes.^13 21^ These actions can often be implemented opportunistically during routine or exacerbation-related consultations. By contrast, initiating or transitioning patients from pMDI to DPI preventers represents a qualitatively different behavioural task. It requires a longer, more deliberate clinical review, including the assessment of inhaler suitability, patient training and prescriber confidence, which may only be feasible during annual reviews. These features introduce significant logistical barriers, which may explain why SABA-related metrics shifted more than preventer inhaler-related metrics during the study.

Although pMDIs may be clinically appropriate in some scenarios, the UK continues to exhibit disproportionately high pMDI use compared with European neighbours (71.6% vs 10%–30%), despite reductions since 2008.^14 15^ Persistent barriers to change have been documented through clinician interviews in the North of England showing that time constraints, burnout and fear of patient refusal are key obstacles, while financial incentives act as motivators.^51^ Patient surveys suggest willingness to switch: 60% of pMDI users were open to changing device type, while 92% cited ease of use, 68% portability, 69% carbon footprint and 51% spacer requirements as influential factors.^52 53^ 65% of UK patients were also unaware of the environmental impact of pMDIs.^53^ A recent qualitative study explored climate-friendly inhaler prescribing using the Theoretical Domains Framework, where they found concerns over healthcare professional knowledge gaps, perceptions of patient preference, uncertainty about clinical equivalence, device-handling concerns, time pressures and organisational inertia as major challenges.^54^ Our intervention may not have been sufficient to overcome these barriers; however, further research is needed to determine what messaging and strategies are most effective in addressing persistent detrimental patterns of preventer and high-carbon inhaler prescribing among both clinicians and patients.

While there has been debate within the implementation sciences field around the need to sacrifice scientific rigour for practical relevance, this trial has shown that it is entirely possible to embed efficient, cost-friendly, rigorous evaluations into healthcare improvement programmes that provide relevant answers to real-world questions.^55^ The University of Leeds, NHS WY ICB and the West Yorkshire NIHR Applied Research Collaboration have effectively developed a ‘Learning Health System’, which uses established data infrastructures to embed A&F research into regional campaigns on high-priority issues.^25 26^ Such learning systems are gaining traction as they offer opportunities for researchers and healthcare systems to conduct embedded, collaborative research, using systematic approaches to iterative, data-driven improvement.^56^ The results presented here further progress the hypothesis that continued, sustainable teamworking through the learning health systems model can efficiently test multiple implementation strategies and target multiple implementation challenges to produce continuous development and learning.

## Conclusion

At a healthcare system level, a significant proportion of the clinical, environmental and social impact of asthma can be directly influenced by clinicians and their prescribing behaviours. Results presented here suggest that paper reports may not be a necessary component of an A&F intervention that also uses digital reports. Challenges remain to understand the barriers to influencing the prescribing of preventer inhalers and moving inhaler devices to low-carbon ‘green’ alternatives. This study has demonstrated the functionality of the learning health system model at scale while establishing that implementation science can use rigorous and efficient trial designs without sacrificing cost-efficiency and real-world relevance.

## Supplementary material

10.1136/bmjresp-2025-003601 online supplemental file 1

## Data Availability

Data are available on reasonable request.

## References

[1] D’Amato G, Cecchi L, D’Amato M (2014). Climate change and respiratory diseases. Eur Respir Rev.

[2] Dyer C (2020). Air pollution from road traffic contributed to girl’s death from asthma, coroner concludes. BMJ.

[3] Vos T, Lim SS, Abbafati C (2020). Global burden of 369 diseases and injuries in 204 countries and territories, 1990–2019: a systematic analysis for the Global Burden of Disease Study 2019. Lancet.

[4] Levy ML (2015). The national review of asthma deaths: what did we learn and what needs to change?. *Breathe (Sheff*).

[5] Levy M, Andrews R, Buckingham R (2014). Why asthma still kills - The national review of asthma deaths (NRAD).

[6] Suissa S, Ernst P, Benayoun S (2000). Low-dose inhaled corticosteroids and the prevention of death from asthma. N Engl J Med.

[7] Vincken W, Levy ML, Scullion J (2018). Spacer devices for inhaled therapy: why use them, and how?. ERJ Open Res.

[8] Laube BL, Janssens HM, de Jongh FHC (2011). What the pulmonary specialist should know about the new inhalation therapies. Eur Respir J.

[9] Ramadan WH, Sarkis AT (2017). Patterns of use of dry powder inhalers versus pressurized metered-dose inhalers devices in adult patients with chronic obstructive pulmonary disease or asthma: An observational comparative study. Chron Respir Dis.

[10] Azzi E, Srour P, Armour C (2017). Practice makes perfect: self-reported adherence a positive marker of inhaler technique maintenance. NPJ Prim Care Resp Med.

[11] Newman SP (1995). A comparison of lung deposition patterns between different asthma inhalers. J Aerosol Med.

[12] Woodcock A, Janson C, Rees J (2022). Effects of switching from a metered dose inhaler to a dry powder inhaler on climate emissions and asthma control: post-hoc analysis. Thorax.

[13] NICE (2024). Asthma: diagnosis, monitoring and chronic asthma management (BTS, NICE, SIGN) (NG245).

[14] Seeley R (2022). Bulletin 295: inhaler carbon footprint.

[15] Lavorini F, Corrigan CJ, Barnes PJ (2011). Retail sales of inhalation devices in European countries: So much for a global policy. Respir Med.

[16] Hancox RJ, Cowan JO, Flannery EM (2000). Bronchodilator tolerance and rebound bronchoconstriction during regular inhaled beta-agonist treatment. Respir Med.

[17] Aldridge RE, Hancox RJ, Robin Taylor D (2000). Effects of terbutaline and budesonide on sputum cells and bronchial hyperresponsiveness in asthma. Am J Respir Crit Care Med.

[18] Bloom CI, Cabrera C, Arnetorp S (2020). Asthma-Related Health Outcomes Associated with Short-Acting β_2_-Agonist Inhaler Use: An Observational UK Study as Part of the SABINA Global Program. Adv Ther.

[19] Suissa S, Ernst P, Boivin JF (1994). A cohort analysis of excess mortality in asthma and the use of inhaled beta-agonists. Am J Respir Crit Care Med.

[20] NHS England (2020). Greener NHS » Delivering a ‘Net Zero’ national health service.

[21] Global Initiative for Asthma (GINA) (2025). Global strategy for asthma management and prevention 2025.

[22] Ivers NM, Sales A, Colquhoun H (2014). No more “business as usual” with audit and feedback interventions: towards an agenda for a reinvigorated intervention. Implement Sci.

[23] Ivers N, Yogasingam S, Lacroix M (2025). Audit and feedback: effects on professional practice. Cochrane Database Syst Rev.

[24] Ivers NM, Grimshaw JM, Jamtvedt G (2014). Growing literature, stagnant science? Systematic review, meta-regression and cumulative analysis of audit and feedback interventions in health care. J Gen Intern Med.

[25] Sheikh A, Anderson M, Albala S (2021). Health information technology and digital innovation for national learning health and care systems. Lancet Digit Health.

[26] Foy R, Skrypak M, Alderson S (2020). Revitalising audit and feedback to improve patient care. BMJ.

[27] Foy R, Willis T, Glidewell L (2020). Developing and evaluating packages to support implementation of quality indicators in general practice: the ASPIRE research programme, including two cluster RCTs. *Programme Grants Appl Res*.

[28] Wood S, Foy R, Willis TA (2021). General practice responses to opioid prescribing feedback: a qualitative process evaluation. Br J Gen Pract.

[29] Salmerón L, Altamura L, Delgado P (2024). Reading comprehension on handheld devices versus on paper: A narrative review and meta-analysis of the medium effect and its moderators. J Educ Psychol.

[30] Annisette LE, Lafreniere KD (2017). Social media, texting, and personality: A test of the shallowing hypothesis. Pers Individ Dif.

[31] Koljonen D (2008). The environmental impact of mail - a baseline.

[32] Alderson SL, Farragher TM, Willis TA (2021). The effects of an evidence- and theory-informed feedback intervention on opioid prescribing for non-cancer pain in primary care: A controlled interrupted time series analysis. PLoS Med.

[33] Hoffmann TC, Glasziou PP, Boutron I (2014). Better reporting of interventions: template for intervention description and replication (TIDieR) checklist and guide. BMJ.

[34] Price D, Chrystyn H, Kaplan A (2012). Effectiveness of Same Versus Mixed Asthma Inhaler Devices: A Retrospective Observational Study in Primary Care. *Allergy Asthma Immunol Res*.

[35] Ministry of Housing, Communities & Local Government (2019). English indices of deprivation 2019.

[36] West Yorkshire Combined Authority (2021). West Yorkshire state of the region report 2021.

[37] Office for Health Improvement and Disparities National general practice profiles - Data - OHID. https://fingertips.phe.org.uk/profile/general-practice/data#page/0/gid/2000005/pat/223/par/E40000013/ati/221/are/nE54000054/iid/639/age/28/sex/4/cat/-1/ctp/-1/yrr/1/cid/4/tbm/1/page-options/ovw-do-0.

[38] Goldstein CE, Taljaard M, Dixon SN (2025). Navigating the consent river: questions to consider before waiving consent requirements in pragmatic cluster randomised trials. *J Med Ethics*.

[39] Baird B, Beech J (2020). Primary care networks explained. https://www.kingsfund.org.uk/publications/primary-care-networks-explained.

[40] (2023). Bennett Institute for applied data science. Home. https://openprescribing.net/.

[41] Office for Health Improvement and Disparities National general practice profiles - OHID. National general practice profiles. https://fingertips.phe.org.uk/profile/general-practice.

[42] Hopewell S, Chan A-W, Collins GS (2023). CONSORT 2025 statement: updated guideline for reporting randomised trials. BMJ.

[43] StataCorp (2023). Stata statistical software.

[44] Royal Mail (2022). Commercial letter products life cycle assessment.

[45] Capstick T, Yousaf A, Hayward-Reed E (2024). The feasibility and impact of implementing a maintenance and reliever (MART) prescribing strategy in Leeds (ID 532).

[46] Willis TA, Collinson M, Glidewell L (2020). An adaptable implementation package targeting evidence-based indicators in primary care: A pragmatic cluster-randomised evaluation. PLoS Med.

[47] Giguère A, Zomahoun HTV, Carmichael P-H (2020). Printed educational materials: effects on professional practice and healthcare outcomes. Cochrane Database Syst Rev.

[48] Baird B, Maguire D (2021). Understanding factors that enabled digital service change in general practice during the COVID-19 pandemic.

[49] Tsimtsiou Z, Fragkoulis E, Koupidis S (2022). Greece: Introducing paperless, remote ePRESCRIPTION— A game-changer for primary care services (2021).

[50] Ivers N, Jamtvedt G, Flottorp S (2012). Audit and feedback: effects on professional practice and healthcare outcomes. Cochrane Database Syst Rev.

[51] Franklin L, Twohig H, Mallen C (2023). Greener asthma prescribing study: a qualitative study exploring healthcare professional perspectives on reducing the prescribing of metered dose inhalers for asthma to reduce the carbon footprint of primary care.

[52] Rothwell E, McElvaney J, Fitzpatrick A (2024). Evaluating inhaler technique, patient preferences and opportunities for improvement in hospitals in the UK. Future Healthc J.

[53] D’Ancona G, Cumella A, Renwick C (2021). The sustainability agenda and inhaled therapy: what do patients want?.

[54] Oosterveld B, Broese JMC, Ossebaard H (2025). Are we ready for climate-friendly inhaler prescription and usage? A qualitative study among primary and secondary care patients, healthcare professionals and healthcare insurers in the Netherlands. BMJ Open.

[55] Geng EH, Peiris D, Kruk ME (2017). Implementation science: Relevance in the real world without sacrificing rigor. PLoS Med.

[56] Grimshaw JM, Ivers N, Linklater S (2019). Reinvigorating stagnant science: implementation laboratories and a meta-laboratory to efficiently advance the science of audit and feedback. BMJ Qual Saf.

