# Supplementary material for: Comparing paper Letters in addition to Emailed Audit and feedback in Refining Asthma treatment to Improve clinical and environmental Results in primary care through a cluster randomised controlled trial: the CLEAR AIR study
Source: BMJ Open Respir Res. 2026 Apr 17;13(1):e003601. doi: 10.1136/bmjresp-2025-003601 (PMC13110629; doi:10.1136/bmjresp-2025-003601)
Supplement: online supplemental file 1 [file bmjresp-13-1-s001.docx]

## **Appendix I – A&F intervention sample:**


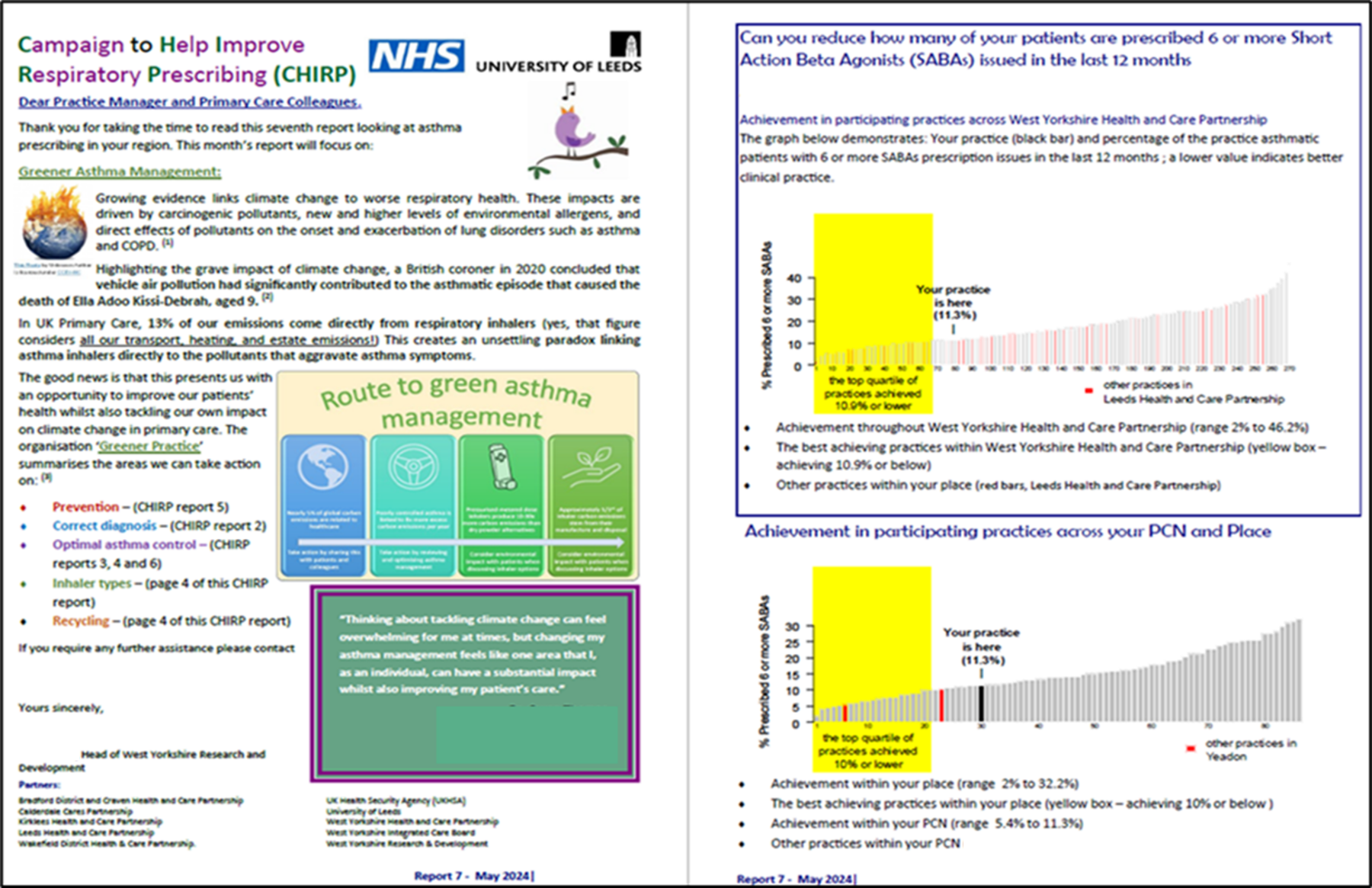


## **Appendix II – TIDieR checklist for the CHIRP Intervention and CLEAR AIR study, incorporating the reporting and design elements of audit and feedback interventions recommendations**

**1. Brief name: Provide the name or a phrase that describes the intervention**

Campaign to Help Improve Respiratory Prescribing (CHIRP) / Comparing paper Letters in addition to Emailed Audit and feedback in Refining Asthma treatment to Improve clinical and environmental Results in primary care (CLEAR AIR)

**2. Why: Describe any rationale, theory, or goal of the elements essential to the intervention**

Audit and feedback (A&F) aims to improve patient care by reviewing health care performance against explicit standards. Ideally, where a discrepancy is detected, changes are implemented at an individual, team, and/or service level. Our earlier work identified the scale of inappropriate prescribing for asthma in primary care, alongside its excessive carbon footprint. Given accumulating evidence of harm from reports such as the UK NRAD report, adjusting current prescribing trends to be more in-line with national and international guidelines is likely to improve patient outcomes, reduced avoidable deaths, and minimise associated carbon emissions.

**3. What (materials): Describe any physical or informational materials used in the intervention, including those provided to participants or used in intervention delivery or in training of intervention providers. Provide information on where the materials can be accessed (for example, online appendix, URL)**

The intervention entailed general practices receiving a comparative and practice-individualised feedback report on the prescribing patterns for patients coded with asthma amongst their practice population. The primary outcome was the proportion of preventer inhalers prescribed that used pMDI devices as a proportion of all available device options. Secondary outcomes included the proportion of patients with asthma that were: using six or more SABAs per year; using twelve or more SABAs per year; using three or less ICS inhalers per year; requiring two or more oral courses of prednisolone per year; aged 0-19 without smoking exposure status recorded; using a mix of inhaler device types.

Aggregated patient data for each practice were extracted from electronic health record systems and reports were sent within two weeks of extraction. Individual prescriber-level data were not available and no patient outcome data were included. The reports presented the data as total patient numbers and percentage of patient population, as well as in a bar chart that highlighted practices in the same area (ICB and PCN) and the lowest quartile of prescribers. Reports incorporated evidence-informed behaviour change techniques – such as specific recommendations for action and an action plan to complete – alongside the 15 recommendations for audit and feedback, which are designed to enhance effectiveness.

**4. What (procedures): Describe each of the procedures, activities, and/or processes used in the intervention, including any enabling or support activities**

In the control group, digital emailed copies were sent to key members of staff at each practice identified by the report distribution group, of which the majority were either the practice manager or GP partner of the GP practice. The intervention group received an email in the same way, but also received physical paper copies sent via post directly to the practice manager.

**5. Who provided: For each category of intervention provider (for example, psychologist, nursing assistant), describe their expertise, background and any specific training given**

Reports were sent by the West Yorkshire Research and Development team on behalf of the West Yorkshire ICB and the research team at the University of Leeds. The reports were written by a primary care registrar and academic clinical fellow in primary care and reviewed / edited by the research team.

**6. How: Describe the modes of delivery (such as face to face or by some other mechanism, such as internet or telephone) of the intervention and whether it was provided individually or in a group**

Practices that received physical paper copies received five copies of the feedback reports sent by post to each practice, addressed to the practice manager, from May 2023 to May 2024 by the research team. Reports were also sent by email to the practice managers or GP Partner depending on practice preference in all intervention groups.

**7. Where: Describe the type(s) of location(s) where the intervention occurred, including any necessary infrastructure or relevant features**

All practices that were recruited received their allocated intervention; However, two practices and three practices merged respectively to form two new practices, leaving 270 practices for analysis.

**8. When and how much: Describe the number of times the intervention was delivered and over what period of time including the number of sessions, their schedule, and their duration, intensity or dose**

Feedback reports delivered bimonthly from May 2023 to May 2024 with a total of seven reports sent.

**9. Tailoring: If the intervention was planned to be personalised, titrated or adapted, then describe what, why, when, and how**

The interventions were not tailored beyond practice’s receiving different statistics based on their performance.

**10. Modifications: If the intervention was modified during the course of the study, describe the changes (what, why, when, and how)**

The intervention was not modified during the course of the study.

**11. How well (planned): If intervention adherence or fidelity was assessed, describe how and by whom, and if any strategies were used to maintain or improve fidelity, describe them**

Intervention adherence and fidelity was not assessed.

**12. How well (actual): If intervention adherence or fidelity was assessed, describe the extent to which the intervention was delivered as planned**

Intervention adherence and fidelity was not assessed.

# **Appendices:**

## **Appendix III – Statistical base code sample (STATA):**

GEE – RR:

xtgeebcv two_pred_base arm_label prac_pop place imd_decile two_pred_outcome, family(binomial) link(log) corr(exchangeable) vce(robust) stderr(kc) cluster(pcn) eform

GLMM REML:

mixed sabaemissions_outcome i.arm_label prac_pop place sabaemissions_base|| pcn:, reml dfmethod(kroger) dftable(default) nolog

GEE OR:

xtgeebcv twelve_saba_outcome arm_label prac_pop place imd_decile twelve_saba_base, family(binomial) link(logit) corr(exchangeable) vce(robust) stderr(kc) cluster(pcn) eform

Wilcoxon signed-rank test:

signrank twelve_saba_base = twelve_saba_outcome

## **Appendix IV – Sensitivity analysis:**


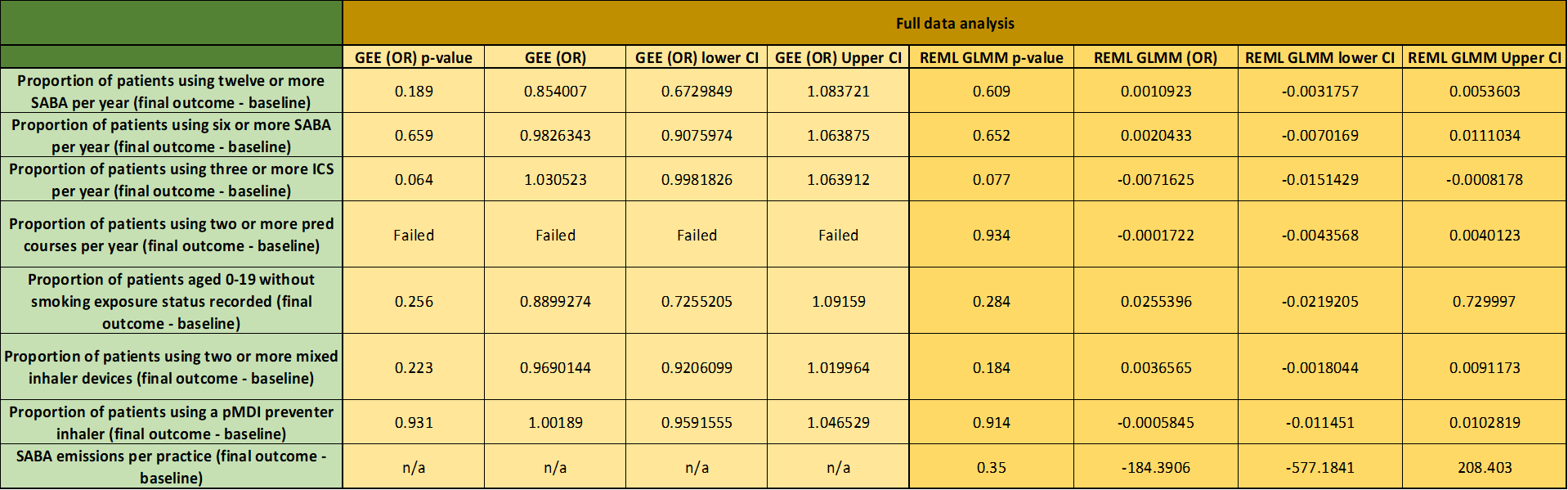


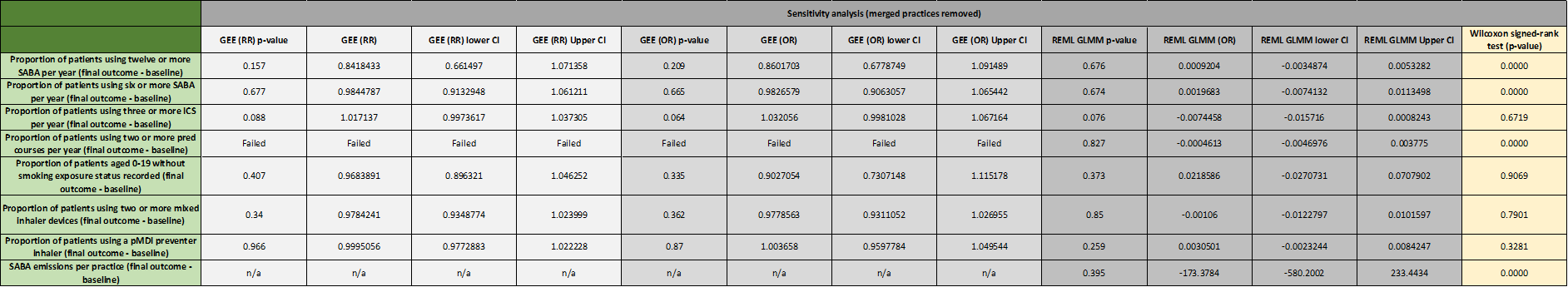


## **
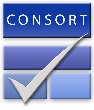
Appendix V – CONSORT checklist and the cRCT CONSORT extension (abstract included):**

CONSORT 2010 checklist – randomised trial with the cluster extension (1,2)

| Section/Topic | Item No | Checklist item | Reported on page No |
| --- | --- | --- | --- |
| Title and abstract | | | |
|  | 1a | Identification as a cluster randomised trial in the title | 1 |
|  | 1b | Structured summary of trial design, methods, results, and conclusions (for specific guidance see CONSORT for abstracts) | 1 |
| Introduction | | | |
| Background and objectives | 2a | Scientific background and explanation of rationale (Rationale for using a cluster design) | 3 & 4 |
|  | 2b | Specific objectives or hypotheses. Whether objectives pertain to the cluster level, the individual participant level, or both | 3 & 4 |
| Methods | | | |
| Trial design | 3a | Description of trial design (such as parallel, factorial) including allocation ratio. Definition of cluster and description of how the design features apply to the clusters | 4 |
|  | 3b | Important changes to methods after trial commencement (such as eligibility criteria), with reasons | 7 |
| Participants | 4a | Eligibility criteria for participants and clusters | 4 |
|  | 4b | Settings and locations where the data were collected | 4 & 5 |
| Interventions | 5 | The interventions for each group with sufficient details to allow replication, including how and when they were actually administered. Whether interventions pertain to the cluster level, the individual participant level, or both. | 4 |
| Outcomes | 6a | Completely defined pre-specified primary and secondary outcome measures, including how and when they were assessed. Whether outcome measures pertain to the cluster level, the individual participant level, or both. | 4 |
|  | 6b | Any changes to trial outcomes after the trial commenced, with reasons | n/a |
| Sample size | 7a | How sample size was determined. Method of calculation, number of clusters(s) (and whether equal or unequal cluster sizes are assumed), cluster size, a coefficient of intracluster correlation (ICC or k), and an indication of its uncertainty | 5 |
|  | 7b | When applicable, explanation of any interim analyses and stopping guidelines | n/a |
| Randomisation: |  |  |  |
| Sequence generation | 8a | Method used to generate the random allocation sequence | 4 |
|  | 8b | Type of randomisation; details of any restriction (such as blocking and block size). Details of stratification or matching if used | 4 |
| Allocation concealment mechanism | 9 | Mechanism used to implement the random allocation sequence (such as sequentially numbered containers), describing any steps taken to conceal the sequence until interventions were assigned. Specification that allocation was based on clusters rather than individuals and whether allocation concealment (if any) was at the cluster level, the individual participant level, or both | 4 |
| Implementation: |  |  |  |
| Implementation | 10a | Who generated the random allocation sequence, who enrolled clusters, and who assigned clusters to interventions | 4 |
| Implementation | 10b | Mechanism by which individual participants were included in clusters for the purposes of the trial (such as complete enumeration, random sampling) | 4 |
| Implementation | 10c | From whom consent was sought (representatives of the cluster, or individual cluster members, or both) and whether consent was sought before or after randomisation | 4 |
| Blinding | 11a | If done, who was blinded after assignment to interventions (for example, participants, care providers, those assessing outcomes) and how | 4 |
|  | 11b | If relevant, description of the similarity of interventions | 4 |
| Statistical methods | 12a | Statistical methods used to compare groups for primary and secondary outcomes. How clustering was taken into account. | 5 |
|  | 12b | Methods for additional analyses, such as subgroup analyses and adjusted analyses | 5 |
| Results | | | |
| Participant flow (a diagram is strongly recommended) | 13a | For each group, the numbers of participants who were randomly assigned, received intended treatment, and were analysed for the primary outcome. For each group, the numbers of clusters that were randomly assigned, received intended treatment, and were analysed for the primary outcome | 7 & 8 |
|  | 13b | For each group, losses and exclusions after randomisation, together with reasons. For each group, losses and exclusions for both clusters and individual cluster members | 7 |
| Recruitment | 14a | Dates defining the periods of recruitment and follow-up | 7 |
|  | 14b | Why the trial ended or was stopped | 7 |
| Baseline data | 15 | A table showing baseline demographic and clinical characteristics for each group. Baseline characteristics for the individual and cluster levels as applicable for each group | 8 |
| Numbers analysed | 16 | For each group, number of participants (denominator) included in each analysis and whether the analysis was by original assigned groups. For each group, number of clusters included in each analysis. | 7 & 8 |
| Outcomes and estimation | 17a | For each primary and secondary outcome, results for each group, and the estimated effect size and its precision (such as 95% confidence interval). Results at the individual or cluster level as applicable and a coefficient of intracluster correlation (ICC or k) for each primary outcome | 9 and N/A |
|  | 17b | For binary outcomes, presentation of both absolute and relative effect sizes is recommended | 9 |
| Ancillary analyses | 18 | Results of any other analyses performed, including subgroup analyses and adjusted analyses, distinguishing pre-specified from exploratory | 10 |
| Harms | 19 | All important harms or unintended effects in each group (for specific guidance see CONSORT for harms) | n/a |
| Discussion | | | |
| Limitations | 20 | Trial limitations, addressing sources of potential bias, imprecision, and, if relevant, multiplicity of analyses | 11 |
| Generalisability | 21 | Generalisability (external validity, applicability) of the trial findings. Generalisability to clusters and/or individual participants (as relevant) | 11 |
| Interpretation | 22 | Interpretation consistent with results, balancing benefits and harms, and considering other relevant evidence | 11 |
| Other information | | |  |
| Registration | 23 | Registration number and name of trial registry | 6 |
| Protocol | 24 | Where the full trial protocol can be accessed, if available | 6 |
| Funding | 25 | Sources of funding and other support (such as supply of drugs), role of funders | 6 |
| **Abstract** |  |  |  |
| Title | 25 | Identification of study as cluster randomised | Abstract |
| Trial design | 27 | Description of the trial design (for example, parallel, cluster, non-inferiority) | Abstract |
| Methods: |  |  |  |
| Participants | 28 | Eligibility criteria for participants and the settings where the data were collected. Eligibility criteria for clusters | Abstract |
| Interventions | 29 | Interventions intended for each group | Abstract |
| Objective | 30 | Specific objective or hypothesis. Whether objective or hypothesis pertains to the cluster level, the individual participant level, or both | Abstract |
| Outcome | 31 | Clearly defined primary outcome for this report. Whether the primary outcome pertains to the cluster level, the individual participant level or both | Abstract |
| Randomisation | 32 | How participants were allocated to interventions. How clusters were allocated to interventions | Abstract |
| Blinding (masking) | 33 | Whether or not participants, care givers, and those assessing the outcomes were blinded to group assignment | Abstract |
| Results: |  |  |  |
| Numbers randomised | 34 | Number of participants randomised to each group. Number of clusters randomised to each group | Abstract |
| Recruitment | 35 | Trial status* | Abstract |
| Numbers analysed | 36 | Number of participants analysed in each group. Number of clusters analysed in each group | Abstract |
| Outcome | 37 | For the primary outcome, a result for each group and the estimated effect size and its precision | Abstract |
| Harms | 38 | Important adverse events or side effects. Results at the cluster or individual level as applicable for each primary outcome | n/a |
| Conclusions | 39 | General interpretation of the results | Abstract |
| Trial registration | 40 | Registration number and name of trial register | Present in main body |
| Funding | 41 | Source of funding | Present in main body |

**Citation:**

1. Schulz KF, Altman DG, Moher D, for the CONSORT Group. CONSORT 2010 Statement: updated guidelines for reporting parallel group randomised trials. BMC Medicine. 2010;8:18.
2. Campbell MK, Piaggio G, Elbourne DR, et al. Consort 2010 statement: extension to cluster randomised trials. BMJ. 2012;345:e5661. doi: 10.1136/bmj.e5661

© 2010 Schulz et al. This is an Open Access article distributed under the terms of the Creative Commons Attribution License (<http://creativecommons.org/licenses/by/2.0>), which permits unrestricted use, distribution, and reproduction in any medium, provided the original work is properly cited.

*We strongly recommend reading this statement in conjunction with the CONSORT 2010 Explanation and Elaboration for important clarifications on all the items. If relevant, we also recommend reading CONSORT extensions for cluster randomised trials, non-inferiority and equivalence trials, non-pharmacological treatments, herbal interventions, and pragmatic trials. Additional extensions are forthcoming: for those and for up-to-date references relevant to this checklist, see [www.consort-statement.org](http://www.consort-statement.org).

## **Appendix VI – Direct additional economic and carbon footprint cost estimated from provided printed reports in addition to virtual reports for 1 year of the CHIRP project in West Yorkshire**

| **Total printing additional cost** | | | | | |
| --- | --- | --- | --- | --- | --- |
| **Activity to produce 1 report** | **Band** | **No. of Hours** | **Cost £** | | **Total for 7 reports (£)** |
| Printing of reports | 6 | 22.5 | 579.12 | | 4053.85722 |
| Print address labels | 6 | 2 | 51.48 | | 360.342864 |
| Staple x 5 reports and put in envelopes | 6 | 15 | 386.08 | | 2702.57148 |
| **Cost of printing (1 report)** | **No. of sheets** | **£** | **VAT £** | **Total £** | **Total for 7 reports (£)** |
| Latest invoice for double sided colour print (DSCP) | 160,878 | 625.00 | 125.00 | 750.00 | 5250 |
| **Paper costs (1 report)** | **No. of sheets** | **£** | **VAT £** | **Total £** | **Total for 7 reports (£)** |
| 1 box of paper = 5 reams 500 sheets per ream | 2,500 | 21.00 | 4.20 | 25.20 | 176.4 |
| **Envelope costs (1 report)** | **No. of envelopes** | **£** | **VAT £** | **Total £** | **Total for 7 reports (£)** |
| 1 box of no window A4 white envelopes | 250 | 13.00 | 2.60 | 15.60 | 109.2 |
| **Staple costs (1 report)** | **No. of staples** | **£** | **VAT £** | **Total £** | **Total for 7 reports (£)** |
| 1 box of standard staples | 5,000 | 0.65 | 0.13 | 0.78 | 5.46 |
| **Address label costs (1 report)** | **No. of labels** | **£** | **VAT £** | **Total £** | **Total for 7 reports** |
| 1 box of labels 14 per sheet, 100 sheets | 1,400 | 34.00 | 6.80 | 40.80 | 285.6 |
| **Postage type (1 report)** | **Costs** | **No. of practices** | **Total £** | | **Total for 7 reports (£)** |
| First class postage | £1.70 | 270 | £459.00 | | 3,213.00 |
| **Overall total** | | | | **£16,156.43** | |
|  |  |  |  |  |  |
| **Total printing carbon footprint additional burden** | | | | | |
| **Royal mail A4 Letter (transactional pack) carbon footprint (gCO₂e)** | **No. of packs per practice** | **No. of practices** | **No of reports** | **Overall carbon footprint (gCO₂e)** | **Overall carbon footprint (KgCO₂e)** |
| 184.36 | 5 | 270 | 7 | 1742202 | **1742.202** |
